# Porokeratosis of Mibelli in an HIV-positive patient*

**DOI:** 10.1590/abd1806-4841.20164253

**Published:** 2016

**Authors:** Luiza de Queiroz Ottoni, Priscila Kakizaki, Rafael Ribeiro Pinheiro, José Alexandre de Souza Sittart, Neusa Yuriko Sakai Valente

**Affiliations:** 1 Private clinic – Belo Horizonte (MG) – Brazil; 2 Hospital do Servidor Público Estadual de São Paulo (HSPE) – São Paulo (SP) – Brazil; 3 Private clinic – São Paulo (SP) – Brazil

**Keywords:** HIV, Immunosuppression, Porokeratosis

## Abstract

Porokeratosis represents a group of disorders of epidermal keratinization that are characterized by one or more annular plaques surrounded by a histologically distinctive hyperkeratotic ridge-like border called the cornoid lamella. Many studies showed that organ transplantation and immunosuppression were associated in a significant number of cases. Furthermore, an association with squamous cell carcinoma and basal cell carcinoma has been noted in all variants of porokeratosis. The rarity of this disorder and its atypical clinical presentation – a single lesion on the thumb of an HIV-positive male patient – motivated this report.

## INTRODUCTION

Porokeratosis (PK) is a group of abnormal keratinization in the epidermis.^1^ It is a chronic and often hereditary (autosomal dominant) disorder. However, most cases appear to be sporadic. Lesions are characterized by annular keratotic papules or plaques with raised peripheral ridges that spread centrifugally. It was first described in 1893 by Mibelli.^2-5^ It is usually asymptomatic, but pruritus may occur.^2,3^ PK variants are associated, and more than one type may develop in a patient. Localized forms include PK of Mibelli (PM), linear PK and punctate PK. Disseminated forms include superficial, actinic superficial, and palmoplantar PK.^1-5^

Lesions may occur in any part of the body including the mucous membranes, but extremities are the most affected sites. Multiple lesions may occur, but are usually unilateral and regionally located.

The etiology of PK is multifactorial and includes genetic factors, ultraviolet radiation, trauma, and infectious agents.^1^ Cases associated with organ transplantation, hematologic malignancies, HIV infection, use of immunosuppressant drugs, and chemotherapy have been reported.^2,3,4^ Some reports suggest a relationship between the lesion evolution and the degree of immunosuppression.^1,6,7^ Clinical and molecular evidence has shown that PK can be considered a premalignant condition.^8^ The rarity of this disorder and its atypical clinical presentation – a single lesion on the thumb of an HIV-positive patient – motivated this report.

## CASE REPORT

**Figure 1:**
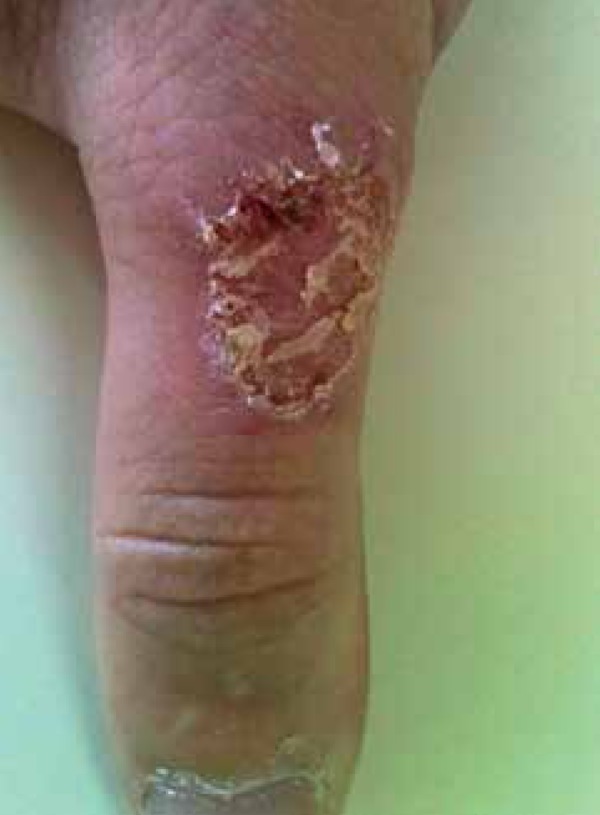
Scaly plaques with raised ridges and an atrophic center on the dorsal surface of the right first digit

**Figure 2:**
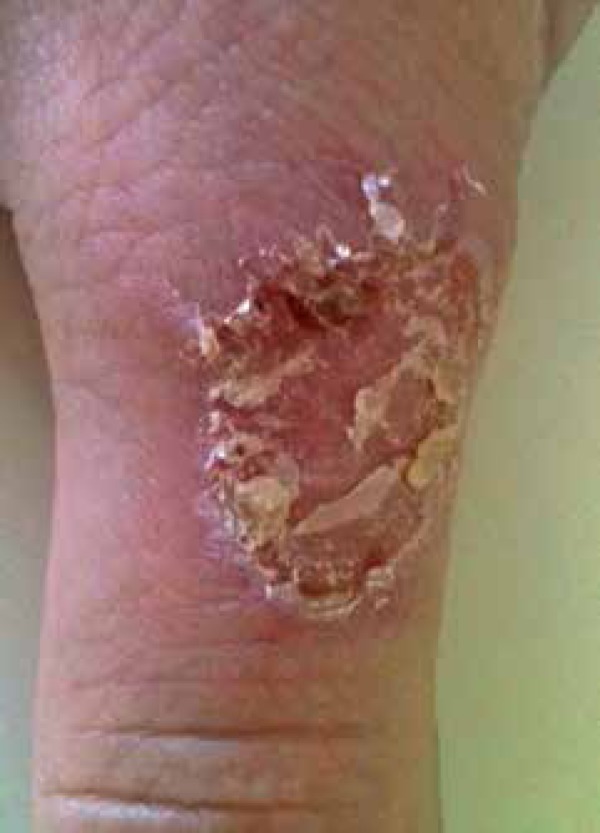
Figure 1 lesion in detail

**Figure 3:**
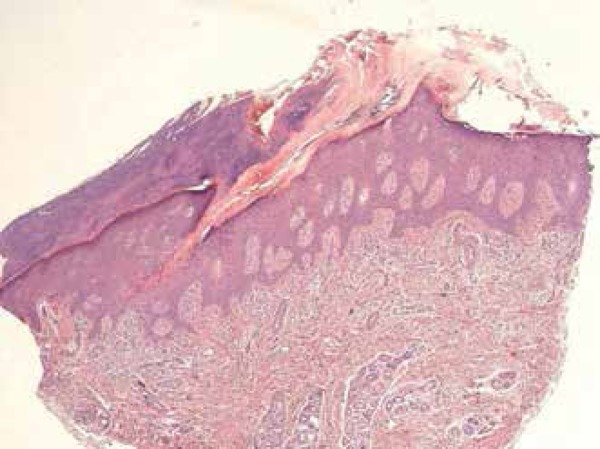
Histopathology showing a cornoid lamella embedded in the epidermis depression

**Figure 4:**
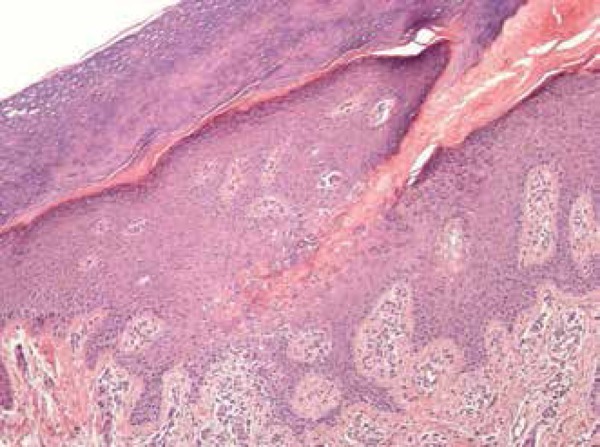
Detail of cornoid lamella, hypogranulosis, and dyskeratosis

**Figure 5:**
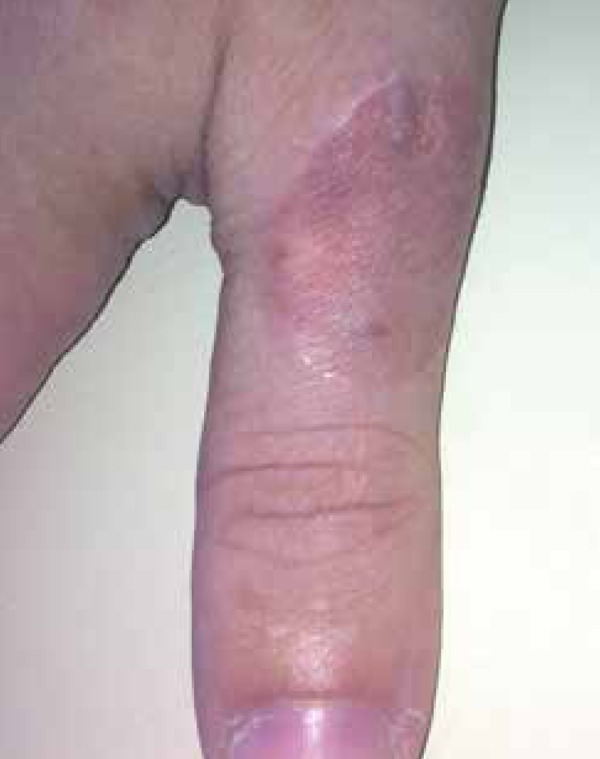
Lesion after excision and reconstruction with graft

We report a white 34-year-old male patient referred to our institution with a one-year lesion on the right first digit. The lesion appeared as an erythematous papule, which markedly progressed in the last six months. The patient reported local pain and bleeding after minor traumas, but denies pruritus. He had been diagnosed with HIV six years before. His latest examinations revealed undetectable viral load and CD4 718 cells/mm^3^. Antiretroviral drugs included tenofovir, lamivudine, lopinavir, and ritonavir. He denied other comorbidities and similar family history. Dermatological examination showed erythematous, keratotic, scaly plaques with raised ridges and an atrophic center on the dorsal surface of the right first digit (Figures 1 and 2). We observed no other lesion. Histopathological examination revealed a large cornoid lamella with parakeratotic cells. Hypogranulosis and dyskeratosis were observed at the base, features suggestive of PM. Superficial dermis showed rare perivascular mononuclear cells (Figures 3 and 4). Considering the possibility of cancer in an immunocompromised patient, we decided to perform a surgical removal of the entire lesion – preserving the functionality of the limb – and to use a skin graft for acceptable esthetics (Figure 5).

## DISCUSSION

PM is a rare entity. Lesion may appear at any age, but are most common during childhood, especially when inherited. The disease has predilection for males.^2-5^ No clear evidence of ethnic predilection has been reported. It often affects the limbs and the involvement of a single digit is unusual. It starts as small and asymptomatic hyperkeratotic skin colored to brownish papules. The lesions can spread over years to form a plaque with raised ridges that can cover an area bigger than 20 centimeters in diameter. The center of the lesions may be hyperpigmented, hypopigmented, atrophic and/or anhidrotic, and occasionally hypertrophic.^2,3,4^ PK dermoscopy shows yellow-brownish cornoid lamellae and linear, globular, or dotted vessels.^1^ Differential diagnoses include: actinic keratosis, stucco keratosis, psoriasis, Darier's disease, basal cell nevus syndrome, among others.^2,5^

The identification of a cornoid lamella is characteristic and essential for the histopathological diagnosis of PK. It corresponds to the hyperkeratotic ridge and is characterized by a thin column of compact parakeratotic cells extending from the invagination of the epidermis to the adjacent skin.^4,5^ PK associated with immunosuppression showed no microscopic characteristic features.^1^ Some reports of PK immunohistochemistry in patients with AIDS showed a near absence of Langerhans cells. Other cases report reduced filaggrin expression and increased involucrin expression.^1,7^

The classical assumption is that PK lesions are due to the expansion of a clone of mutant epidermal keratinocytes located at the base of the cornoid lamella. In addition to the genetic predisposition (instability of chromosome 3p12-14 and mutation of the mevalonate kinase gene -MVK), abnormal proliferation may be triggered by radiation, infectious agents, trauma, and immunosuppression.^1-6^ The association of PK and immunosuppression was observed by Mac-Millan and Roberts in 1974.^3^ After the observation of PK development in a renal transplant patient, different associations have been reported: different types of organ transplants, hematologic malignancies, HIV infection, and inflammatory or autoimmune diseases treated with immunosuppressants. In many cases, PK course was parallel to the current level of immunosuppression, and the lesions occasionally regressed upon discontinuation of the immunosuppressive medication.^1,4,6^ Changes in function of local or systemic immunity may affect immunosurveillance, allowing for the proliferation of clones of mutated keratinocytes.^2,3,4,6^

Late sporadic cases have been linked to drugs, such as thiazide diuretics, and biologics, such as etanercept in patients with psoriasis.^2^ The latency period between the onset of immunosuppressive therapy and the appearance of PK ranged from 1 week to 16 years.^3^

Malignant degeneration has been reported in all forms of PK with a 7.5-11% incidence.^1,2^ Squamous and basal cell carcinoma and Bowen’s disease may occur in PK lesions, the former being most common.^5^ Risk factors include extensive lesions located on the extremities and prolonged evolution period.^1,2,8^ The oncogenic potential may be the result of an increased p53 expression in the keratinocytes near the cornoid lamella.^2,5^

PK treatment can be done with topical medications, such as keratolytic, 5-fluorouracil 5%, retinoids, imiquimod, diclofenac, vitamin D derivatives, and cantharidin tacrolimus.^1^ Cryotherapy, photodynamic therapy, dermabrasion, excision, CO_2_laser, and other lasers can be used with varying degrees of success. In disseminated or refractory lesions, oral retinoid may be beneficial despite the recurrence after discontinuation of the therapy.^1,5^ Surgery is the most effective treatment.^1,8^ In our case – considering that the patient had a local lesion and the possibility of cancer as reported by the literature – we opted to perform surgical removal of the entire lesion and to use a skin graft with excellent functional and aesthetic results for the patient. We point out that, although PK is not considered indicative of AIDS, its appearance or presence in HIV-infected patients could be an immunodeficiency marker.
